# Supplementary material for: Antifungal and Antibiofilm Activity of Cyclic Temporin L Peptide Analogues against Albicans and Non-Albicans Candida Species
Source: Pharmaceutics. 2022 Feb 21;14(2):454. doi: 10.3390/pharmaceutics14020454 (PMC8877061; doi:10.3390/pharmaceutics14020454)
Supplement: Supplementary file 1 [file pharmaceutics-14-00454-s001.zip › pharmaceutics-1575508-supplementary.pdf]

pharmaceutics

Supplementary Materials: Antifungal and Antibiofilm Activity of Cyclic Temporin L Peptide Analogues against Albicans and Non-Albicans *Candida* Species

Rosa Bellavita, Angela Maione, Francesco Merlino, Antonietta Siciliano, Principia Dardano, Luca De Stefano, Stefania Galdiero, Emilia Galdiero, Paolo Grieco and Annarita Falanga

Table of contents

1. Analytical data of peptides 3, 7, 15 and 16 (Table S1)	S2
2. HPLC chromatograms of peptides 3,7, 15 and 16 (Figure S1–4)	S2

Table S1. Analytical data for peptides **3, 7, 15** and **16.**

ID	t_R (min)	Molecular formula	$[M+H]^+_{calcd}$	$[M+2H]^+/2_{obs}$
**3**	13.9	$C_{89}H_{131}N_{20}O_{14}^+$	1704.0148	853.0122
**7**	11.3	$C_{79}H_{117}N_{22}O_{14}^+$	1597.9114	799.9601
**15**	13.9	$C_{89}H_{131}N_{20}O_{14}^+$	1704.0148	853.0122
**16**	14.2	$C_{84}H_{122}N_{19}O_{13}S_2^+$	1668.8905	835.8973

2. HPLC chromatograms of peptides 3,7, 15 and 16

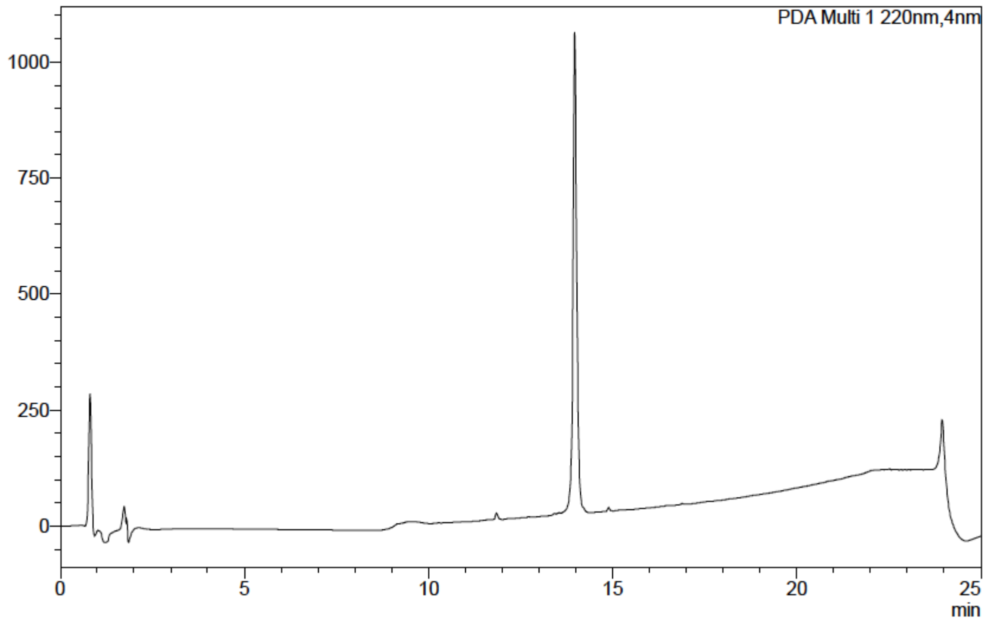

Figure S1. Chromatogram of lactam-bridged peptide **3** obtained by an analytical HPLC (Shimadzu UFLC SPD-M20A/DGU-20A3R/LC-20AD) equipped with a Phenomenex Kinetex C18 column (150 mm × 4.6 mm, 5 µm, 100 Å), and monitored by UV detection at 220 nm. t_R: 13.9 min [linear gradient 10-90% MeCN (0.1% TFA) in H_2O (0.1% TFA) over 20 min, flow rate of 1 mL/min].

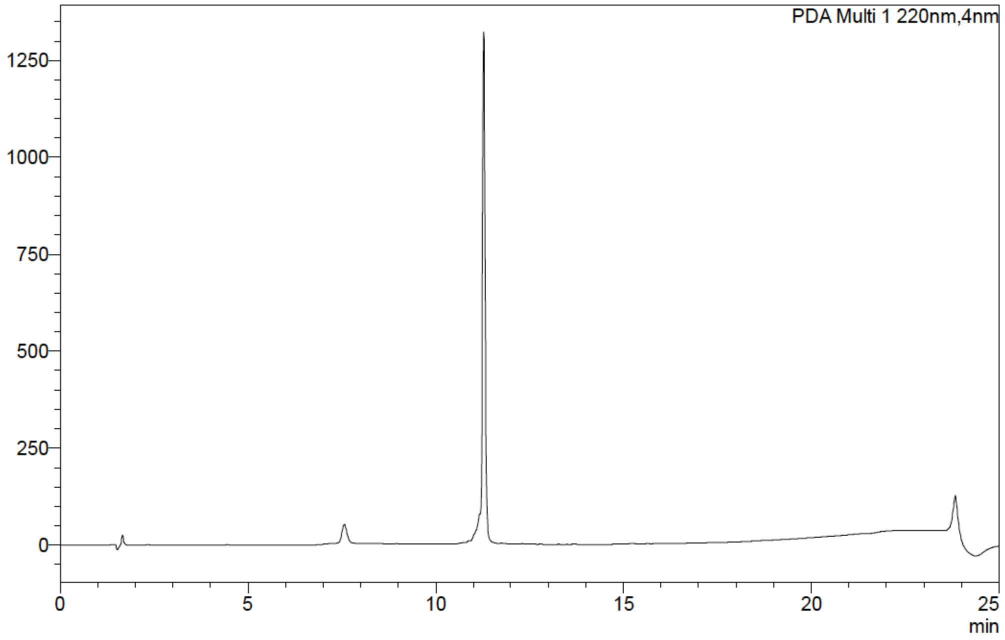

Figure S2. Chromatogram of 1,4-triazolic- bridged peptide **7** obtained by an analytical HPLC (Shimadzu UFLC SPD-M20A/DGU-20A3R/LC-20AD) equipped with a Phenomenex Kinetex C18 column (150 mm × 4.6 mm, 5 μm, 100 Å), and monitored by UV detection at 220 nm. t_R:11.3 min [linear gradient 10–90% MeCN (0.1% TFA) in H_2O (0.1% TFA) over 20 min, flow rate of 1 mL/min].

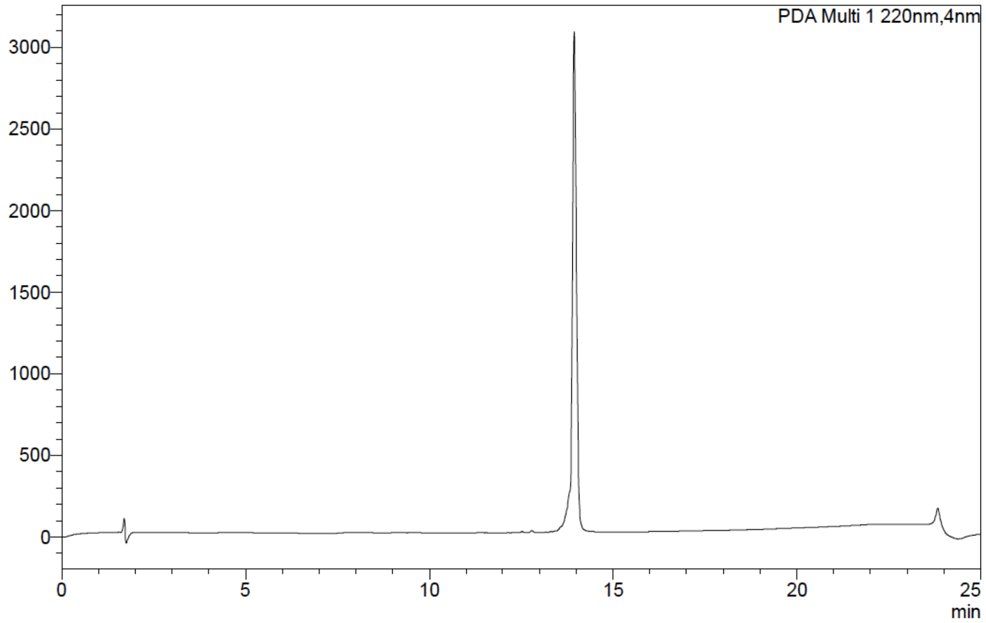

Figure S3. Chromatogram of lactam-bridged peptide **15** obtained by an analytical HPLC (Shimadzu UFLC SPD-M20A/DGU-20A3R/LC-20AD) equipped with a Phenomenex Kinetex C18 column (150 mm × 4.6 mm, 5 μm, 100 Å), and monitored by UV detection at 220 nm. t_R: 13.9 min [linear gradient 10–90% MeCN (0.1% TFA) in H_2O (0.1% TFA) over 20 min, flow rate of 1 mL/min].

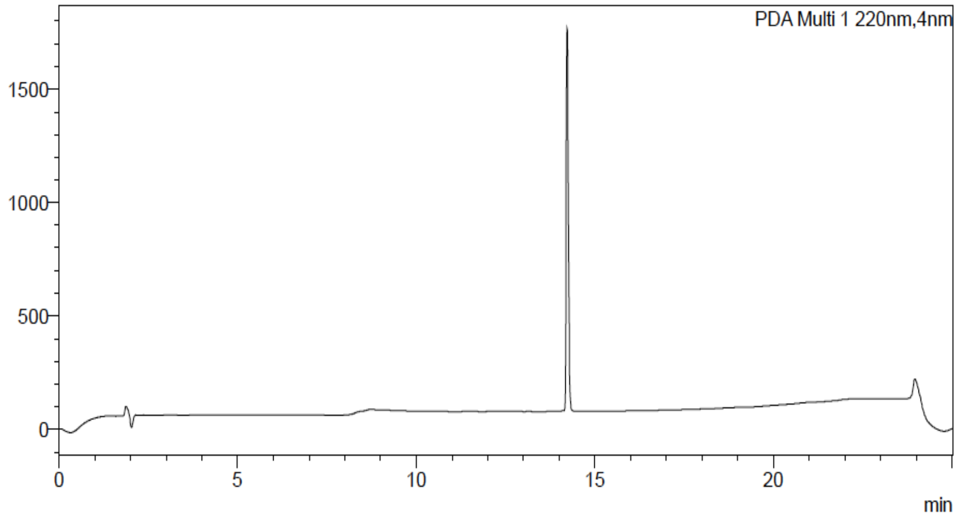

Figure S4. Chromatogram of disulfide-bridged peptide **16** obtained by an analytical HPLC (Shimadzu UFLC SPD-M20A/DGU-20A3R/LC-20AD) equipped with a Phenomenex Kinetex C18 column (150 mm × 4.6 mm, 5 μm, 100 Å), and monitored by UV detection at 220 nm. t_R: 14.2 min [linear gradient 10–90% MeCN (0.1% TFA) in H_2O (0.1% TFA) over 20 min, flow rate of 1 mL/min].
